# Functional Prediction of Candidate MicroRNAs for CRC Management Using in Silico Approach

**DOI:** 10.3390/ijms20205190

**Published:** 2019-10-19

**Authors:** Adewale Oluwaseun Fadaka, Ashley Pretorius, Ashwil Klein

**Affiliations:** Department of Biotechnology, Faculty of Natural Sciences, University of the Western Cape, Private Bag X17, Bellville, Cape Town 7535, South Africa; aspretorius@uwc.ac.za (A.P.); aklein@uwc.ac.za (A.K.)

**Keywords:** microRNA, CRC, functional predictions, in silico analysis, triplex binding site

## Abstract

Approximately 30–50% of malignant growths can be prevented by avoiding risk factors and implementing evidence-based strategies. Colorectal cancer (CRC) accounted for the second most common cancer and the third most common cause of cancer death worldwide. This cancer subtype can be reduced by early detection and patients’ management. In this study, the functional roles of the identified microRNAs were determined using an in silico pipeline. Five microRNAs identified using an in silico approach alongside their seven target genes from our previous study were used as datasets in this study. Furthermore, the secondary structure and the thermodynamic energies of the microRNAs were revealed by Mfold algorithm. The triplex binding ability of the oligonucleotide with the target promoters were analyzed by Trident. Finally, evolutionary stage-specific somatic events and co-expression analysis of the target genes in CRC were analyzed by SEECancer and GeneMANIA plugin in Cytoscape. Four of the five microRNAs have the potential to form more than one secondary structure. The ranges of the observed/expected ratio of CpG dinucleotides of these genes range from 0.60 to 1.22. Three of the candidate microRNA were capable of forming multiple triplexes along with three of the target mRNAs. Four of the total targets were involved in either early or metastatic stage-specific events while three other genes were either a product of antecedent or subsequent events of the four genes implicated in CRC. The secondary structure of the candidate microRNAs can be used to explain the different degrees of genetic regulation in CRC due to their conformational role to modulate target interaction. Furthermore, due to the regulation of important genes in the CRC pathway and the enrichment of the microRNA with triplex binding sites, they may be a useful diagnostic biomarker for the disease subtype.

## 1. Introduction

With increasing incidence and mortality of cancers, colorectal cancer (CRC) remains one of the leading malignant cancers [1]. Currently, tumor resection followed by chemotherapy is one of the most effective treatments for CRC but recurrences are inevitable [2]. Approximately 45% of recurrences are in the first year after initial tumour removal, and over 90% of recurrences occur within four years [3]. In CRC, staging and pathological characteristics are one of the major predictors of diagnosis to enhance treatment options [4]. Thus far, there are limited biomarkers to predict CRC. The search for more biomarkers that are specific for staging is required.

Recent research suggests that microRNAs regulate many gene functions in human cancers [5], and these oligonucleotide sequences have been proposed as novel biomarkers for cancers [6]. Previous studies have shown that their expressions are altered in numerous types of cancers including CRC [7,8]. They also act as regulators of gene expression [9] through gene repression and/or mRNA degradation in many biological processes such as apoptosis, cell development, cell differentiation, cell proliferation, and metabolism [10,11,12]. These biological processes are crucial in carcinogenesis [13]. Although several studies have reported an association between microRNAs and CRC development [14,15,16,17], the role of microRNAs forming triplexes with their targets to infer function in CRC has been barely explored. Several microRNAs have been reported to date but their laboratory functional determination in relation to CRC is challenging and time-consuming.

In silico approaches have been developed to connect sequences of microRNAs and their targets to infer function in Cancer studies. Biologists are mainly particular about the structural and functional properties of any newly derived sequence (protein or nucleotide). In silico predictions are therefore important for this discovery based on successful knowledge-based principles. These principles rely on the fact that the best way to predict the structure and/ or function is to find similar sequences in existing databases using the information about them to infer conclusions about properties of the new sequence. Algorithms have been developed and implemented as computer programs (local or web-based tools) to perform this function.

The genomic sequence of many higher eukaryotes is now complete, and the patterns of expression of thousands of genes under diverse conditions are known. This offers researchers the opportunity to identify and analyze the parts of a genome believed to be responsible for most transcription control, known as the promoters [18]. Short nucleotide sequences, most especially non-coding RNAs, have been reportedly used in the induction of DNA cleavage at specific sites with the aid of triplex formation between the RNA and the DNA [19,20]. The various triplex formation between these nucleotides has suggested a role in gene expression regulation [21,22]. The binding of DNA leading to the triplex bond formation through a hydrogen bond to the third strand has been experimentally discovered but the biological functions are still unknown [23]. The interaction of microRNAs with DNA have not been studied extensively [24,25]. Reports also suggested that the interactions between microRNAs and gene promoter regions may play a more direct role in regulating the efficiency of transcription of the gene involved [26,27,28].

One of the roles includes the methylation of CpG islands [29]. However, other mechanisms yet unidentified or not fully studied could be possible for the direct interaction of microRNAs with genes for transcription regulation. Since dsDNA is capable of forming triplex structures through interactions with DNA or RNA in the major groove of the DNA duplex, researchers postulated that microRNA may form triplex structures with duplex DNA through either Hoogsteen or reverse Hoogsteen hydrogen bonds, and thereby directly interacting with target DNA sequences in regulatory regions including gene promoters in the human genome, with the potential to alter gene function [30,31,32]. Bioinformatics approaches revealed that the mammalian genome is composed of several triplex formation binding sites [24,30,33,34]. This suggests that tethering RNA to specific genomic sites might guide RNA-associated regulatory proteins to establish an epigenetic landscape that facilitates or inhibits gene expression. In this study, the function of the candidate microRNAs was predicted for CRC management using an in silico approach. Given the ever-expanding number of microRNAs, understanding their functional aspects through sequences represents a promising research area.

## 2. Results

### Secondary Structure of the Candidate MicroRNAs

The function of a given microRNA molecule may be determined by sequence or structure that it is most likely to fold into. It may also be governed by whether small sequence have the ability to fold into a particular substructure even if the substructure does not appear in any optimal fold.

## 3. Discussion

Genes implicated as targets for the candidate microRNAs have been experimentally validated and their associations with CRC confirmed by previous studies [35,36,37,38,39,40,41,42,43]. Also, research has confirmed that the regulators of these targets have been experimentally determined and validated in other cancer subtypes but not in CRC [44,45,46,47,48,49,50,51,52,53,54]. Therefore, the interaction of both the microRNAs and targets could suggest their involvement in CRC. The prognostic, diagnostic, and expression analysis performed on these genes together with the gene ontology analysis from our previous study suggest that they could be used as good diagnostic and therapeutic biomarkers in the detection and management of CRC.

### 3.1. Secondary Structure and the Thermodynamic Energies of the Candidate MicroRNAs

**Figure 1:**
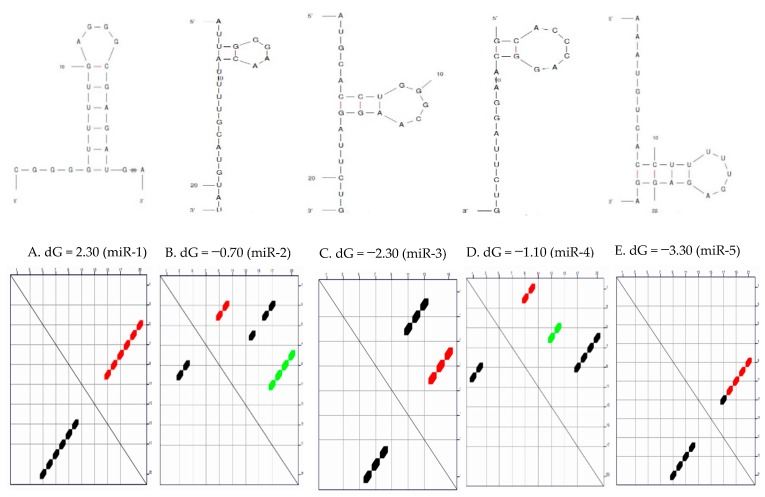
The predicted secondary structure of the five candidate microRNAs with their dot plot directly below showing the optimal energy. (**A**). microRNA miR-1 with its optimal energy of −2.30; (**B**). MicroRNA miR-2 with its optimal energy of −0.70; (**C**). microRNA miR-3 with its optimal energy of −2.30; (**D**). microRNA miR-4 with its optimal energy of −1.10; (**E**). microRNA miR-5 with its optimal energy of −3.30. For the dot plots, red, black, and green dots represent all the optimal foldings (superposition of all possible sub-optimal foldings) Therefore, each colour represents a potential folding configuration.

MicroRNAs have intrinsic potential to form secondary and tertiary structures by folding through base pairing. This folding is given primarily by the fact that certain nucleotides have an affinity for binding another nucleotide, for example (G-C and A-U/). Each nucleotide can only bind with one partner at a time and long stretches of rich nucleotide as a binding partner elsewhere on the sequence are favoured over short stretches or individual non-sequential pairing. These pairings compete with each other since any G in the sequence could pair with any C and similarly, any A with any U. The arrangement in Figure 1 may be optimal in terms of maximizing the length of the concurrent run and pair of nucleotides. These arrangements are only probabilistic and outside forces such as temperature and pH may induce a different pairing arrangement. The molecule may even transition randomly between numerous newly optimal structures based on the fluctuation in available energy. The mature sequences of the five candidate microRNAs were subjected to secondary structure prediction by Mfold and inspected manually against filtering criteria, as indicated by default to check for any discrepancies.

The prediction of microRNA secondary structure is a long-established problem of computational biology which has received a lot of attention in recent years due to mounting evidence that underscores the significant of microRNA structure in a wide variety of biological processes [55,56,57]. The results showed that four of the five have the potential to form more than one secondary structure (folding configuration). Studies have reported numerous microRNAs as biomarkers of disease and potential therapeutic targets due to the fact that their secondary structures may give insight into orchestrated microRNA-dependent gene regulation and be a step forward to understand their functions and involvement in carcinogenesis, and improve therapeutic designs. The results obtained from the secondary structure prediction of the candidate microRNAs are consistent with previous in silico structural determination studies and have indicated that over 70% of human microRNAs may fold into a hairpin structure and almost 70% could potentially form self-aggregated homoduplexes [58,59,60]. All the predicted microRNAs showed hairpin structure which could be enhanced by the cellular environment.

Valid prediction of microRNA–mRNA binding energies is crucial for the understanding of interactions. The thermodynamics of microRNA interactions can be understood as the sum of the energy necessary to ‘open’ the binding site and the energy gained from hybridization [57]. The thermodynamic parameters of the secondary structure of the candidate microRNAs are represented in Table 1 above. Ronchieri et al. [61] reported that microRNA secondary structures contribute to target recognition because there is an energetic cost to freeing base-pairing interactions within mRNA in order to make the target accessible for microRNA binding [62,63,64]. The ΔG values of the candidate microRNAs were calculated using Mfold (Figure 1 and Table 1). The optimal sequences were predicted to have optimal folding in miR-1 and 5, while the sub-optimal sequences were predicted to have sub-optimal folding in miR-2 and 4. Understanding the optimal folding of base pairs is the least likely secondary structure formation during the reaction [65]. The lower the free energy the more stability of the microRNA. From Table 1, miR-5 is the most stable microRNA, followed by miR-3 and miR-1, respectively.

### 3.2. CpG Island of the Promoter Sequences

The phenomenon of tumor alteration through epigenetic silencing associated with dense hypermethylation of CpG islands, and their complex interplay with modifications in histone structure, provides an alternate mechanism to genetic inactivation of tumor suppressor genes via loss or mutation [66]. Binding sites in the genome have a great regulatory impact on the gene activities in their neighborhood. Predictive tools are therefore essential for deciphering the overall regulatory potential of gene control regions such as promoters and enhancers. The CpG island analysis of the promoters of the prioritized microRNA target genes (7 genes) is shown in Table 2 with Min. GC% of 51% observed in APC while Max. GC% of 83% was observed in KRAS. The ranges of the observed/expected ratio of CpG dinucleotides of these genes are from 0.60 to 1.22. CpG islands have been reported to be found in approximately 50% of human promoters [67,68]. Promoters with CpG islands are associated with housekeeping genes [69,70] and are identified by three primary characteristics: (1) they are over 200 base pairs in length, (2) they have over 50% GC composition, and (3) they retain an observed/expected ratio of CpG dinucleotides greater than 0.6 [71]. In normal tissues, CpG islands associated with tumor suppressor genes are unmethylated but during tumor formation, they are often methylated. Evidence suggests that *de novo* methylation of CpG islands induces the silencing of associated tumor suppressor genes and may, in fact, be an important step in tumor formation [72,73]. The particular genes that are hypermethylated in tumor cells are strongly specific to the tissue of origin of the tumor [74]. This analysis, therefore, strengthens the proof that the sequences extracted from the genomes of these genes may be a true promoter sequence.

### 3.3. Triplex Binding Interaction of the MicroRNAs and Target Genes

**Figure 2:**
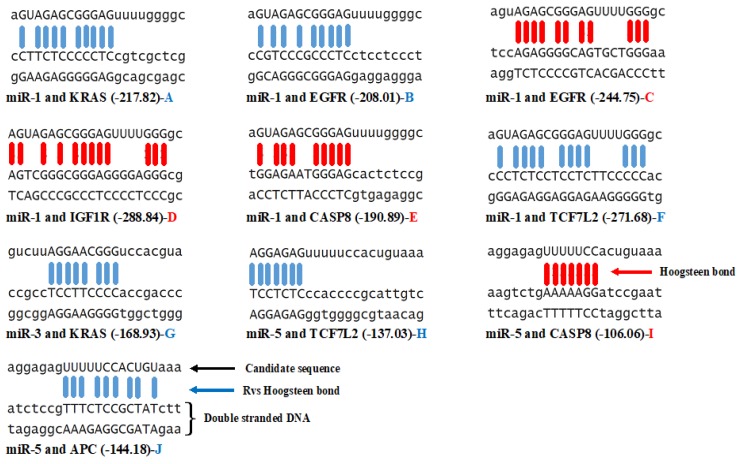
Structural determination of microRNA-DNA triplex formation. The first sequence in each structure (**A**–**J**) represent the candidate microRNA sequences involved in triplex binding while the two sequences without bond are the promoter sequence of the target gene. The blue bond indicates the indirect or reverse hoogsteen bond while the red bond is the direct or hoogsteen bond between the DNA and the microRNA. The negative values in the bracket are the hit energy of each binding.

The specific binding properties of microRNA to proteins or (mRNA) involved in chromatin remodeling and several transcriptional regulations as broadly achieved in the literature demonstrate their multi-functionality [75]. The ability of RNA to participate in triplex formation according to hoogsteen base pairing rules is a less studied property of microRNA [76]. In this study, the binding of the candidate microRNAs to the TSS (600 bp upstream) of the promoter region of the target genes were determined by an in silico analysis using the Trident software. Pasquier et al. [76] reported that formative genes are profoundly represented in the TTS, which certifies the speculation of unexpected large-scale genome regulation mediated by the triplex DNA-RNA structure. The binding of microRNAs to mRNA directly for gene expression regulation has been experimentally validated. However, research has suggested the mechanism by which microRNA forms triplex with double-stranded DNA but their exact mechanism of interaction is less well understood [26,27,77]. Also, Blanco and Montoya [78] elucidated the transient DNA/RNA protein interactions but proposed a further study of triplex formation. From the triplex-forming oligonucleotide formation result represented in Table 3, it was observed that only three of the candidate microRNAs (miR-1, miR-2, and miR-5) are capable of forming multiple triplexes along with three of the target mRNAs (KRAS, TCF7L2, and EGFR). For miR-1, four binding sites were observed in KRAS, and EGFR, while nine binding sites were determined in TCF7L2. This microRNA (miR-1) reports no hit score for the genes APC and GNAS. MicroRNAs 2 and 4 reported no triplex interaction between the target genes showing negative correlation. In microRNA-3, only one reverse (indirect) hoogsteen pairing was observed with KRAS with hit energy of -168.93 (Figure 2). MicroRNA-5 showed two different triplex binding sites in TCF7L2 and one in both APC (indirect) and CASP8 (direct) (Figure 2). All the binding interactions in this study are of grade 5 (99 percentiles of triplex-forming interactions). Both the hit score and hit energy observed are greater than 140 and −140 respectively (Figure 2).

From previous studies, it was recorded that microRNA with a 22-nucleotides sequence forms triplex structures with duplex DNA as documented by fluorescence resonance energy transfer, FRET, surface Plasmon resonance, electromobility shift assay, and Nuclear magnetic resonance [79,80]. Similar to transient protein–protein and DNA/RNA-protein interactions, Blanco and Montoya [78] suggested that transient formation of microRNA-duplex DNA triplexes may have as much biological importance as more stable interactions. Surprisingly, there are enzymes of the class helicase that possess the ability to unwound the intramolecular triplex DNA [30,81], it is feasible that this mode of action of these enzymes is similitude to which microRNA can mediate transcriptional activation [82]. On a whole, the formation of triplexes between the candidate microRNA (1, 3, and 5) and DNA double-stranded suggest that they are well conserved and crucial mechanism of transcription regulation. Comparing the thermodynamic energies of folding and triplex formation (Table 1 and Figure 2), it was observed that these microRNAs are better favoured for triplex binding to regulate gene expression in the cancer subtype than microRNA folding.

### 3.4. Somatic Event Evolution of the MicroRNA Target Genes

**Figure 3:**
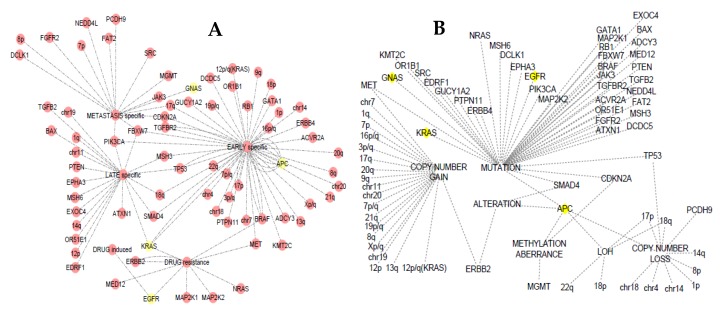
Evolutionary stage-specific somatic events in colorectal cancer (CRC). (**A**) stage-specific targets. (**B**) variant types and specific variants of the microRNA target genes. Yellow nodes indicate the microRNA target genes.

The evolutionary stage-specific and variant events of the microRNA target genes are presented in Figure 3a,b. Using the SEECancer database, all the genes involved in CRC initiation, progression, and metastasis were graphically represented and visualized using Cytoscape. From the network, four (KRAS, GNAS, APC, and EGRF) of the statistically significant genes targeted by the candidate microRNAs were indicated with yellow node. The database also reported that the other three genes (TCF7L2, CASP8, and IGF1R) are either a product of antecedent or subsequent event of the four genes implicated in CRC. TCF7L2 was seen from the database to be a subsequent event of APC mutation. Loss of heterozygosity (LOS) in the APC was shown to affect TP53 gene through mutation which in turn results in the mutation of TCF7L2. APC was found to be specific to early stage of CRC while GNAS was seen at the metastatic stage. Figure 3b shows that alteration, mutation, methylation aberrance, and loss of copy number of APC gene may result in CRC initiation, progression, or metastasis. Previous studies have reported that the loss of function, as well as methylation aberrance and mutation of the APC gene, is associated with early events in CRC [83,84,85,86,87,88,89,90,91,92].

The antecedent event resulting in the mutation of KRAS was also detected to be from the mutation of CASP8 which in turn mutates PIK3CA gene. Mutation in the gene KRAS was revealed to be either at the early, late, or drug resistance stage. Burmer et al. [93] reported the early event of KRAS in CRC as detected by DNA amplification and oligonucleotide hybridization, as well as by RNase A mismatch cleavage analysis. Using a bioinformatics approach, Youn and Simon [94] also suggested that KRAS mutations occur as the first event with high probability for both colorectal and lung tumors. Genome-editing technology also revealed that mutations in this gene are sufficient to initiate tumour progression [95]. The report of Fumagalli et al. [96] using a high throughput experiment suggest that the initiating APC and KRAS mutations drives efficient proliferation and growth, whereas inactivating mutations in SMAD4 block differentiation during tumor progression. Mutation in EGFR was shown to be at the drug-induced and resistance stage event (Figure 3a,b). In metastatic CRC using high throughput experiment, Morelli et al. [97], concluded that acquired mutations of this gene in metastatic CRC patients were correlated with acquired resistance to anti-EGFR monoclonal antibodies. Mutation in other genes targeted by the candidate microRNAs may be involved in colorectal carcinogenesis.

### 3.5. Co-expression Analysis

**Figure 4:**
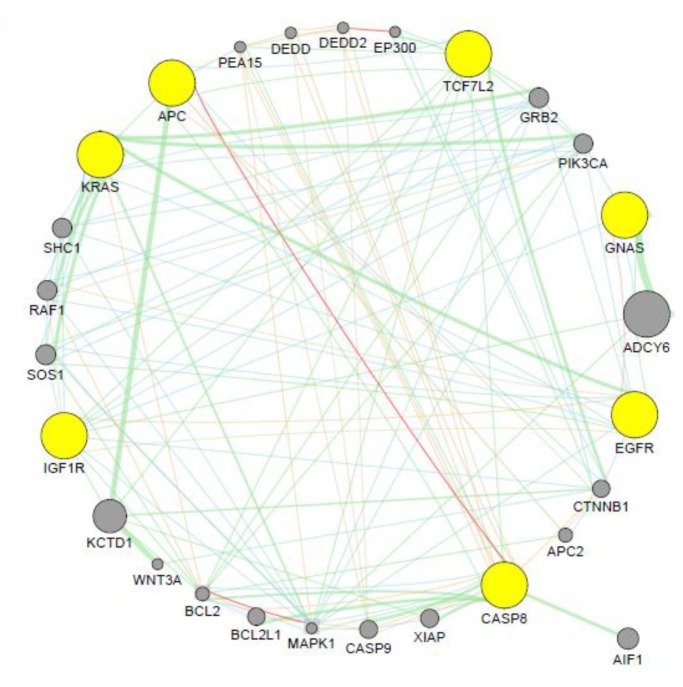
Co-expression analysis network of the prioritized microRNA target genes. The target genes are shown in yellow nodes while other genes in grey represent the associated genes. The red edges showed that the connected genes are co-expressed while the green edges are associated with genetic interactions. Other interactions include shared protein domains, physical interaction, pathway, and predicted interaction.

GeneMANIA was used to generate a hypothesis about the candidate microRNA target genes’ significance. The co-expression analysis of the candidate microRNAs result was represented in Figure 4. The output generated genes that are functionally similar, or have shared properties with microRNA target genes, displayed an interactive functional association network, illustrating the relationships among the genes and gene of interest.

Genetic interaction of this network accounted for 43.83% of the total interactions, shared protein domain accounted for 26.28, physical interaction accounted for 14.23, co-expressed genes accounted for 10.14%. Only 3.89 and 2.62% are accounted for pathway and predicted interaction respectively. Within the microRNA target genes, only APC and CASP8 are co-expressed, although neighbouring genes found to be co-expressed are Bcl-2 and MAPK-1. Since this tool assigns a weighting feature to dataset to determine how genes in a list are well connected to each other or to determine which types of functional genomic data are most useful to retrieve for finding more genes similar to the query, KCTD1 and ADCY6 were assigned the highest weight due to size and are genetically connected with APC and GNAS respectively. Upregulation of ADCY6 activates the CREB pathway by increasing the tumorigenic potential of cells reported in gastric cancer [98]. Certain genes in the Wnt pathway influence KCTD1-mediated downregulation of β-catenin and suggested that KCTD1 functions as a novel inhibitor of Wnt signaling pathway by enhancing β-catenin degradation [99]. Inappropriate activation of this pathway has been observed in CRC [100,101]. These associated genes may then provide further a complete microRNA-gene network for CRC diagnosis and disease management.

## 4. Materials and Methods

The secondary structure, CpG island and triplex binding analysis, Somatic Events in Evolution of the target genes, and co-expression analysis were performed on both the candidate microRNAs and their targets using the following tools: Mfold at http://unafold.rna.albany.edu/?q=mfold, SMS at http://www.bioinformatics.org/sms2/translate.html, NCBI at https://blast.ncbi.nlm.nih.gov/Blast.cgi, Ensembl at http://www.ensembl.org/index.html, UCSC at https://genome.ucsc.edu/, Trident at http://trident.stjude.org/, SEECancer at http://biocc.hrbmu.edu.cn/SEECancer/index.jsp, GeneMANIA at http://genemania.org/, and Cytoscape at https://cytoscape.org/.

### 4.1. Datasets

Five candidate microRNAs decoded as miR-1 to 5 were identified using in silico approach and their targets genes were prioritized through three different target prediction tools (TargetScan, miRDB, and miRDIP) to generate seven genes namely: *APC*, *GNAS*, *EGFR*, *TCF7L2*, *KRAS*, *IGF1R*, and *CASP8*. The sequences of these microRNAs together with the promoter sequences of their targets were used for functional determination in this study.

### 4.2. Structural Determination of Candidate microRNA

The mature sequences of candidate microRNAs were submitted to Mfold program accessed at http://www.mfold.rna.albany.edu for predicting their secondary structure (s) and the free energy. MFold accepts a single nucleotide sequence as input mainly in FASTA format. The output file produced by MFold contains the calculated energy matrices that determine all optimal and suboptimal secondary structures for the folded nucleic acid molecule. This file is read by the companion program, PlotFold, which can display any of several different graphic representations of optimal and suboptimal secondary structures for the folded molecule.

### 4.3. Promoter Sequence Extraction

The database for Ensembl was accessed at http://www.ensembl.org/index.html. The seven prioritized microRNA target genes were used as input to generate their promoter sequences. From the display, the exons are highlighted in the pink background and red text, the sequence before exon 1 is the promoter sequence. Six-hundred base pairs 5′-flanking sequences were retrieved. To prevent disparity of the annotation concerning the transcription start site among databases, the obtained sequences were further analyzed in USCC using the BLAT tool at https://genome-euro.ucsc.edu/. Furthermore, CpG island feature and GC content were considered.

### 4.4. CpG Island Analysis

The sequence manipulator suites (SMS) report potential CpG island regions using the method described by [102]. The calculation was performed using a 200 base pairs window moving across the sequence at 1 base pairs intervals. CpG islands are defined as sequence ranges where the observed/expected value is greater than 0.6 and the GC content is greater than 50%. The expected number of CpG dimers in a window is calculated as the number of ‘C’s in the window multiplied by the number of ‘G’s in the window, divided by the window length. While the ration of the observed to the expected is calculated as Obs/Exp CpG = Number of CpG * N/(Number of C * Number of G), where N = length of the sequence. The CpG island suite was accessed at http://www.bioinformatics.org/sms2/cpg_islands.html for the identification of the CpG islands in the promoter sequences of the prioritized microRNA target genes.

### 4.5. Triplex Binding Analysis

Trident was accessed at http://trident.stjude.org/. This online prediction tool was used to identify triplex binding sites between the candidate microRNAs obtained in our previous study and the promoter region of the statistical significant target genes. Sequences of microRNAs and their targets were used as input. The output of this search was given based on the number of sites that the sequence of the candidate microRNA was able to bind on the promoter sequence. The grade, hit score, types of binding (direct or indirect), and hit energy were also considered.

### 4.6. Staging Analysis

The SEECancer database was accessed at http://biocc.hrbmu.edu.cn/SEECancer to explore the evolutionary stage-specific somatic events in CRC as well as their temporal order [103]. From the web interface, evolutionary stage and temporal order were selected individually to query the target genes as well as the cancer subtype respectively. The result of these search generated a txt file as input in Cytoscape to generate a network of events based on variant types and evolutionary events in CRC.

### 4.7. Co-expression Analysis

A co-expression network identifies which genes have a tendency to show a coordinated expression pattern across a group of samples. This co-expression network can be represented as a gene–gene similarity matrix, which can be used in downstream analyses [104]. Seven prioritized genes target by candidate microRNA were used as input to generate the co-expressed genes using GeneMANIA http://www.genemania.org (http://apps.cytoscape.org/apps/genemania) plugin of Cytoscape. This database is used to generate hypotheses about gene function, analyzing gene lists and prioritizing genes for functional assays. The output is a network of genes with several interactions.

### 4.8. Statistical Analysis

For microRNA selection, the BlastN parameters were set at 1e-2 for expected value, 7.0 for word size, and 90–99% for similarity index. The CD-HIT-EST-2D parameters were set at 0.90 for threshold and 7.0 for word size. The genes considered in DAVID v6.8 were regarded statistically significant at *p*-value of 1.8 × 10^3^ with the Benjamini score of 1.6 × 10^−2^. A value of *p* < 0.05 was considered to indicate a statistically significant difference.

### 4.9. Data Availability 

The datasets and the clinical data were obtained from the online databases as described above in the methods and their websites are as follows: http://www.mirbase.org/ for reference dataset, microRNA associated with CRC at cancer were http://www.picb.ac.cn/dbDEMC/, http://www.mir2disease.org/, http://www.cuilab.cn/hmdd, and http://mircancer.ecu.edu/. While Mfold, SMS, NCBI, Ensembl, UCSC, Trident, SEECancer, GeneMANIA and Cytoscape were accessed from the server.

## 5. Conclusions

Understanding microRNA’s secondary structures, thermodynamic parameters, and targets may deliver greater promise towards their diagnostic potentials and mechanisms in the management of CRC. The secondary structure together with the thermodynamic parameters of the candidate microRNAs may, therefore, provide a valid result regarding their target even when the conservation of the microRNA is unknown. Also, the secondary structure of the candidate microRNAs suggests a conformational role to modulate target interactions and therefore can be used to explain the different degree of genetic regulation in CRC.

MicroRNAs that are capable of triplex formation with duplex DNA are more frequently positively correlated with gene transcripts. MiR-1, miR-3, and miR-5 are suggested to have significant enrichment of positive correlation with the target gene involved in the triplex structure. This analysis further confirmed that the targets of the candidate microRNAs are enriched with microRNA triplex binding sites. Furthermore, microRNA function may then depend not only on their sequences but also their structures and triplex binding interaction with their targets. Hypothetically speaking, microRNAs targeting these genes can be inferred to be an important regulator in the stage-specific events. Since miR-1, miR-2, and miR-3 are regulators of APC, it can be concluded that these microRNAs may be used as a diagnostic tool in the early detection of CRC. Also, miR-1 regulating GNAS may be involved in metastatic stage-specific event in CRC. MicroRNAs regulating KRAS may also be involved in early, late, or drug-resistant stage-specific events in CRC (MiR-1,3 and 5). However, further molecular experiments are on-going to confirm the function of these identified microRNAs alongside their targets in CRC.

## Figures and Tables

**Table 1:** Parameters and secondary structures of the microRNA sequences.

S/N	MicroRNAs	Length	δG (*kcal*/*mol*)	Initial ΔG (*kcal*/*mol*)	Stru^C^
1	miR-1	22	0.0	−2.30	1
2	miR-2	22	0.7	−0.70 −0.40 0.00	3
3	miR-3	22	0.7	−2.80 −2.10	2
4	miR-4	20	0.8	−1.10 −0.80 −0.30	3
5	miR-5	22	0.6	−3.30 −2.70	2

δG: Free energy in plot profile; ΔG: Optimal energy of secondary structures (kcal/mol) at 37 °C with optimal and sub-optimal structures, respectively. Stru^C^: Number of secondary structure calculated by Mfold.

**Table 2:** Result of the CpG island assessed by sequence manipulator suite.

S/N	Gene_ID	Min. GC%	Max. GC%	Min. obs/exp	Max. obs/exp
1	APC	51.00	57.50	0.61	0.67
2	KRAS	59.50	83.00	0.78	1.14
3	TCF7L2	53.00	72.50	0.60	1.00
4	EGFR	56.00	57.00	0.62	0.90
5	IGF1R	53.00	81.50	0.61	1.22
6	CASP8	67.50	70.50	0.60	0.74
7	GNAS	67.50	70.50	0.61	0.78

Note: Min. GC%- Minimum GC content detected in each region of 1-200 base pairs; Max. GC%- Maximum GC content detected in each region of 1-200 base pairs. Min. obs/exp-Minimum observed/expected ratio of CpG dinucleotides. Max. obs/exp- Maximum observed/expected ratio of CpG dinucleotides.

**Table 3:** Output of the results of trident showing different binding sites.

MicroRNA/Gene	KRAS	TCF7L2	APC	EGFR	CASP8	IGF1R	GNAS
miR-1	−4	−9	0	−3/+1	+1	+1	0
miR-2	0	0	0	0	0	0	0
miR-3	−1	0	0	0	0	0	0
miR-4	0	0	0	0	0	0	0
miR-5	0	−2	−1	0	+1	0	0

Legend: (0); No hit, (+); Direct Hoogsteen, (−); Reverse Hoogsteen, (Values); Number of hits; Grade: 5. Hit score: > 140; Hit energy: < −140. The heuristic score (hit score) represents Hoogsteen or Reverse Hoogsteen base pair complementarity and Thermodynamic Energy (hit energy) represents the binding energy of the triplex. The binding sites were categorized based on the number of hits relative to score and energy.

## Abbreviations

miRNA	microRNA
CRC	Colorectal cancer
MRNA	Target genes
δG	Free enengy in plot profile
ΔG	Optinal energy of the secondery structure
